# Efficacy of topically administered fluralaner or imidacloprid/moxidectin on dogs with generalised demodicosis

**DOI:** 10.1186/s13071-018-3230-9

**Published:** 2019-01-25

**Authors:** Josephus J. Fourie, Leon Meyer, Emmanuel Thomas

**Affiliations:** 1 Clinvet International (Pty) Ltd, Uitzich Road, Bainsvlei, Bloemfontein, South Africa; 2 Clinvet Morocco, Douar Dbabej, Beni Yekhlef, 28815 Mohammedia, Morocco; 3 MSD Animal Health Innovation GmbH, GmbH, Zur Propstei, 55270 Schwabenheim, Germany

**Keywords:** Bravecto^TM^ Spot-on Solution, Fluralaner, Advocate®, Spot-on, Imidacloprid, Moxidectin, Efficacy, Dogs, Generalised demodicosis, *Demodex canis*, Mange

## Abstract

**Background:**

Canine demodicosis is classified as localised or generalised according to the extent of the disease. Chronic generalised demodicosis is a difficult skin disease to treat and unlikely to resolve without therapy. This laboratory study compared the efficacy of two topical spot-on medications, fluralaner or a combination of imidacloprid and moxidectin, against naturally acquired generalised demodicosis in dogs.

**Methods:**

Sixteen client-owned dogs with naturally acquired generalised demodicosis were randomly allocated to 1 of 2 study groups consisting of 8 dogs each. On Day 0, dogs in 1 group were treated once with fluralaner spot-on solution. Dogs in the other group were treated with the imidacloprid/moxidectin spot-on solution on 3 occasions (Days 0, 28 and 56) or weekly in severe cases. Mites were counted in skin scrapings and demodectic lesions were evaluated on each dog before treatment, and at 28-day intervals over the 12-week period. Deep skin scrapings were made from the same 5 sites on each dog at each examination.

**Results:**

After administration of fluralaner, miticidal efficacy was 99.7% at Day 28, > 99.9% at Day 56 and 100% at Day 84. Efficacy in dogs treated topically with the imidacloprid and moxidectin combination, was 9.8% at Day 28, 45.4% at Day 56 and 0% at Day 84, and was significantly (*P* < 0.01) lower than the fluralaner treated group at each post-treatment time point.

**Conclusions:**

A single topical administration of fluralaner eliminated *Demodex* sp. mites on dogs with generalised demodicosis. Topical imidacloprid/moxidectin combination treatment administered 3 times at 28-day intervals, or more frequently, did not eliminate mites from most treated dogs.

## Background

A single dose of oral fluralaner is highly effective against a variety of mite species in dogs including *Demodex canis* [1–3]. A new formulation of topically administered fluralaner (Bravecto™ Spot-on Solution, Merck Animal Health, Madison, NJ, USA) is now available. This new option for treating dogs with generalised demodicosis was compared with a topical combination of imidacloprid and moxidectin (Advocate®, Bayer Animal Health, Leverkusen, Germany).

## Methods

This study followed the same methodology previously used to evaluate the efficacy of oral fluralaner and topical imidacloprid and moxidectin against naturally acquired (i.e. not experimentally infected) generalised demodicosis in dogs [1]. Signs of generalised demodicosis were defined as more than 5 affected areas or pododemodicosis involving 2 or more feet, or an entire body region. This was also the primary inclusion criterion for dogs that participated in the study. Other inclusion criteria were: (i) older than 8 weeks; (ii) acclimatisation to the study site for at least 7 days; (iii) deep skin scrapings performed on Day -2 confirmed the presence of *Demodex* spp. mites; (iv) clinically healthy, except for clinical signs and symptoms associated with generalised demodicosis, as evaluated on Day -7 and again on Day -2; (v) not clinically pregnant; (vi) not treated with a glucocorticoid therapy or any ectoparasiticide or macrocyclic lactone for at least 12 weeks prior to Day 0, as far as could be reasonably established by verbal communication during the lease agreement of the animals; (vii) not excessively fractious in that they posed a danger to themselves or facility personnel.

Dogs that did not comply with the inclusion criteria were not allocated to study groups. Sixteen client-owned dogs with naturally acquired generalised demodicosis were, with the owners’ consent, randomly allocated to 1 of 2 study groups consisting of 8 dogs each. Dog age was confirmed as older than 8 weeks (one of the inclusion criteria) by veterinary examination as owners could not provide exact birth dates. All dogs had their permanent denture and the estimated age of every dog was between 6 and 12 months. Randomisation was performed using mite counts prior to treatment as ranking criterion, before using MS Excel software to randomly allocate dogs to the respective groups. Enrolled dogs were transferred to the study site, individually housed indoors, and fed a standard commercially available dry dog food once daily with drinking water provided ad lib.

On the day of treatment, all 8 dogs in 1 group were treated once topically with fluralaner at the minimum label recommended dose of 25 mg fluralaner/kg body weight (BW). This treatment was not repeated on any of the dogs in this group. All 8 dogs in the other group were treated topically with a combination of imidacloprid and moxidectin at the label recommended dose of ≥ 10 mg imidacloprid/kg and 2.5 mg moxidectin/kg BW and on 2 more occasions at monthly intervals (Days 28 and 56). In addition, 4 severely affected dogs, as evaluated by a blinded (no access to group allocation codes) veterinarian, were treated weekly with the imidacloprid and moxidectin combination as recommended on the product prescribing directions [4]. The attending veterinarian considered clinical signs (crusts, casts, scales and erythematous papules) and hair loss within the context of the overall clinical picture of each dog. The veterinarian classified cases as mild, moderate or severe based on professional unbiased and objective (unaware of treatment allocations) opinion of the overall clinical condition of each dog. This severity classification, together with mite counts, were used to guide decisions on monthly or weekly treatments.

All dogs were observed daily for general health with clinical examination by a veterinarian every 2 weeks. All dogs were also treated with an appropriate antibiotic (Convenia®, Zoetis, Whippany, NJ, USA) for potential pyoderma starting 7 days before the topical ectoparasite treatment. Convenia® was selected for precautionary concomitant treatment as it is registered for the treatment of secondary superficial pyoderma. Biopsies were performed on Day -7 to evaluate potential existing cases of pyoderma, and again on Day 27 to confirm the absence of inflammatory cells and bacteria. Parameters assessed during the histopathological examinations were *Demodex*, acanthosis, hyperkeratosis, surface crusting, pigmentary incontinence, follicular keratosis, mural folliculitis, perifolliculitis, dermatitis, bacteria, granulomas and dermal stromal reaction. Convenia® was administered subcutaneously to all dogs at a dose of 0.1ml/kg on Days -7, 7, 21, 35 and 49 (at which time Day 27 biopsy results were available and negative and Convenia® treatment ceased). All dogs also received a probiotic (Protexin® Soluble, Kyron Laboratories, Benrose, South Africa) at least twice weekly. Diagnosis and treatment of potential pyoderma was thus not part of the objective to evaluate effective treatment of generalised demodicosis (live mite counts was the primary criterion), but was employed as a precautionary measure and as an ethical consideration to provide relief to study dogs. Mites were counted in skin scrapings and demodectic lesions were evaluated on each dog before the initial ectoparasiticidal treatment and at 28-day intervals thereafter over the 12 week study period (i.e. lesions were assessed on Days -2, 28, 56 and 84 with counts performed on the latter three occasions). Lesions assessed included erythema, casts, scales, crusts (all expressed as percentage of dogs per group affected) and area(s) of hair loss. The latter was assessed according to a scoring system (score 1, 0 to 50%; score 2, > 50 to ≤ 90%; score 3, > 90%). Overall changes in clinical appearance were also illustrated by pre- and post-administration photographs taken from each dog, showing the overall extent and resolution of demodectic lesions for each dog. All mite counts were performed by site personnel masked to the treatment status of each study dog. Deep skin scrapings (~4 cm^2^) were made from the same 5 sites on each dog at each examination. Scrapings were transferred to a labelled microscope slide containing mineral oil, and examined with the aid of a stereo microscope. All live mites (irrespective of developmental stage) were counted and a single live mite count recorded. Dead mites were not included in the counts.

The average percentage reduction in mite counts from pre- to each post-administration time point for each group was calculated as:

$$ \mathrm{Decrease}\;\left(\%\right)\;\left(\mathrm{group}\right)=\left(\left[\mathrm{M}\ \mathrm{Pre}\hbox{-} \mathrm{administration}-\mathrm{M}\ \mathrm{Post}\hbox{-} \mathrm{administration}\right]/\mathrm{M}\ \mathrm{Pre}\hbox{-} \mathrm{administration}\right)\times 100 $$

where M Pre-administration is the arithmetic mean of the pre-administration live mite counts, and M Post-administration is the arithmetic mean of the post-administration live mite counts.

No specific criteria defining treatment failure or success were applicable, since the treatment efficacy was not based on a success rate per animal, but rather based on the mean reduction of live mite counts (pre- and post-administration) for the specific group as a primary pre-defined criterion. Furthermore, as the primary objective for this study was evaluation of efficacy using live mite counts as a criterion, specific underlying causes leading to canine generalised demodicosis were not investigated.

Mite counts measured during the study were compared using a repeated measures analysis of covariance (RMANCOVA - mixed linear model), with treatment, visit and the interaction of treatment by visit as fixed effects; animal as the random effect; and pre-administration values as covariate (SAS version 9.3 TS Level 1M2, SAS Institute (Pty.) Ltd., Houghton Johannesburg, South Africa). The significance level of the formal tests was 5%, and all tests were two sided.

The covariance structure in the repeated measures analysis was investigated using 4 structural assumptions, namely compound symmetry (CS), CS heterogeneous (CSH), first order autoregressive [AR(1)] and heterogeneous first order autoregressive [ARH(1)]. The assumption giving the minimum value of the Akaike information criterion (AIC) was selected in the final analysis. If the treatment by visit interaction was significant (*P* < 0.05), then the treatment group effect was determined for each visit using the LSMEANS having TRT × VISIT in the statement. If the treatment by visit interaction was not significant, then the main treatment effect was evaluated (using an alpha of 0.05 as significant). Heterogeneous variances in the untransformed mite count data (arithmetic mean), led to the use of a Kruskal-Wallis test instead of a RMANCOVA.

## Results

No treatment-related adverse events were recorded for any dog in the study. Mite counts on treated dogs and calculated efficacy (Table 1)

**Table 1:** Mite counts for each dog and calculated efficacy following treatment of dogs with generalised demodicosis with either topical fluralaner or a combination of imidacloprid and moxidectin

Treatment	Dog	Mite count			
		Baseline (Day -2)	Day 28	Day 56	Day 84
Fluralaner	1	114	0	0	0
	2	159	1	1	0
	3	48	0	0	0
	4	698	0	0	0
	5	240	3	0	0
	6	136	0	0	0
	7	26	0	0	0
	8	72	0	0	0
	Mean	186.6	0.5	0.1	0.0
	Efficacy (%)	na	99.7	> 99.9	100
Imidacloprid /moxidectin	1	36	1	0	5
	2^a^	101	40	1	0
	3^a^	286	144	15	14
	4^a^	165	394	39	5
	5	83	1	95	143
	6	130	24	2	0
	7	308	167	9	1
	8^a^	39	264	466	983
	Mean	143.5	129.4	78.4	143.9
	Efficacy (%)	na	9.8	45.4	0
	*P*-value (Kruskal-Wallis test)	na	*χ*^2^ = 9.8401, *df* = 1, *P* = 0.0017	*χ*^2^ = 9.1891, *df* = 1, *P* = 0.0024	*χ*^2^ = 8.4375, *df* = 1, *P* = 0.0037

^a^Dogs which received a weekly treatment

*Abbreviation*: na not applicable

**Fig. 1:**
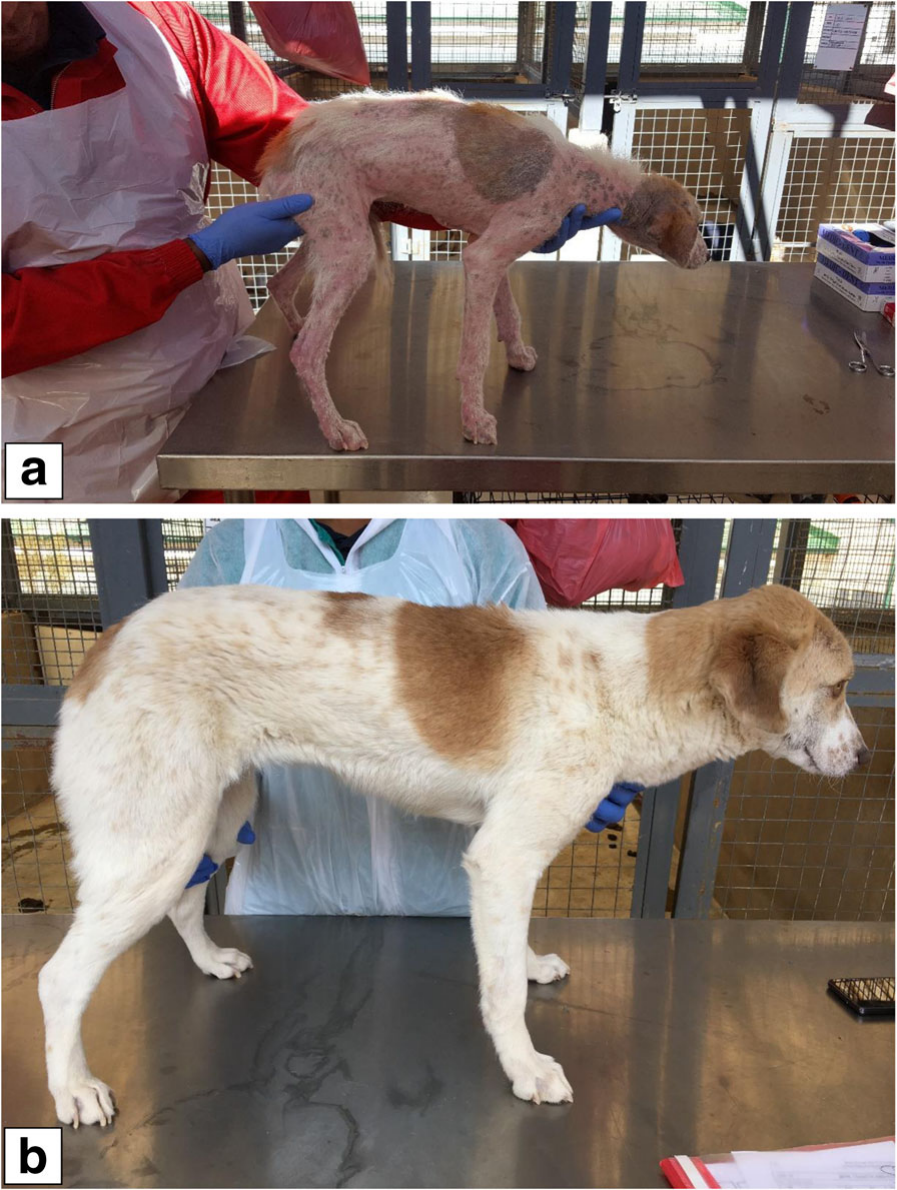
Example of hair re-growth in a dog suffering from generalised demodicosis pre-treatment (**a**) and 12 weeks after treatment with topical fluralaner (**b**)

show that fluralaner treatment was significantly (*P* < 0.01) more effective than the combination of imidacloprid and moxidectin for eliminating mites from dogs with generalised demodicosis at all 3 assessment time points (also see photographic example in Fig. 1)

. In the fluralaner treated group, only 1 dog had 1 mite at 56 days after treatment and there were no mites counted on any of the dogs at 84 days after treatment. Of the dogs treated with a topical combination of imidacloprid and moxidectin, only 1 dog was mite free at 56 days (after at least 2 treatments) and only 2 of 8 dogs were mite free at the end of the 12 week study. However, 2 dogs in the imidacloprid/moxidectin group did show an increase of mite counts after treatment, and although the efficacy was based on the mean mite counts for the group, this impacted significantly on the efficacy for this treatment (refer to the individual mite counts tabulated in Table 1).

## Discussion

These results show that topical fluralaner is highly effective at control of *Demodex* sp. mites in dogs with generalised demodicosis as was previously reported for orally administered fluralaner [1]. The combination of imidacloprid and moxidectin is significantly less effective at eliminating mites. At the final assessment in this study, there was no difference between the mean number of mites on imdicaloprid and moxidectin treated dogs compared to that at the start of the study, primarily because of increasing mite counts on two dogs in this group. These findings are also consistent with previous results [1].

Pharmacokinetic data on orally and topically administered fluralaner show that there are subtle differences between the plasma drug levels following administration by these differing routes. [5, 6]. However, results of the present and previous [1] studies show that either administration approach (topical or oral) provides drug levels sufficient to achieve mite elimination. At the same time, it is interesting that an investigation into mite levels on healthy dogs treated with fluralaner did not find a significant effect on mite populations at the end of the study period [7]. One possible explanation could be that there is a difference in drug levels achieved in hair follicles in dogs with generalised demodicosis compared to the hair follicles of the unaffected dog. However, the above must be considered with caution, as the referenced study [7] evaluated the presence of mite DNA in clinically healthy animals, and not from skin scrapings of visually affected areas.

## Conclusions

A single topical administration of fluralaner is highly effective for eliminating mites from dogs with generalised demodicosis. Repeat topical administrations of a combination of imidacloprid and moxidectin is significantly less effective for the elimination of mites.

## Abbreviations

AIC: Akaike information criterion
AR1: First order autoregressive
ARH1: Heterogeneous first order autoregressive
BW: Body weight
CS: Compound symmetry
CSH: CS heterogeneous
Day: Study day
LSMEANS: Least-squares means
RMANCOVA: Repeated measures analysis of covariance
TRT: Treatment
